# Omarigliptin inhibits brain cell ferroptosis after intracerebral hemorrhage

**DOI:** 10.1038/s41598-023-41635-y

**Published:** 2023-09-01

**Authors:** Yan Zhang, Yang Liu, V. Wee Yong, Mengzhou Xue

**Affiliations:** 1 Department of Cerebrovascular Diseases, The Second Affiliated Hospital of Zhengzhou University, Zhengzhou, 450001 Henan China; 2 Academy of Medical Science, Zhengzhou University, Zhengzhou, Henan China; 3 Hotchkiss Brain Institute and Department of Clinical Neurosciences, University of Calgary, Calgary, AB Canada

**Keywords:** Neuroscience, Cell death in the nervous system

## Abstract

Intracerebral hemorrhage (ICH) is a disastrous disease without effective treatment. An extensive body of evidence indicate that neuronal ferroptosis is a key contributor to neurological disfunctions after ICH. Omarigliptin, also known as MK3102, is an anti-diabetic drug that inhibits dipeptidyl peptidase (DPP4). Recently, MK3102 is reported to exhibit anti-ferroptosis and anti-oxidative effects in different pathological conditions. However, the anti-ferroptosis ability of MK3102 in ICH injury is unknown. Hemin was administrated to model ICH injury in cultured primary cortical neurons, and collagenase VII was used to induce ICH in C57BL/6 mice. MK3102 was administered after ICH. Cell Counting Kit-8 (CCK-8) was applied to detect cell viability. Neurological functions were assessed through the Focal deficits neurological scores and corner test. HE and TUNEL staining was applied to evaluate brain damage areas and cell death, respectively. Ferroptosis was evaluated in cultured neurons by fluorescent probe DCFH-DA, FerroOrange, Liperfluo and immunofluorescence of GPX4, AIFM2 and FACL4. Perls staining was performed to visualize Fe^3+^ deposition. Ferroptosis-related proteins in mouse brain were measured by immunohistochemistry and western blotting. MK3102 reduced the neurotoxicity of hemin in cultured primary cortical neurons. It improved neurological functions associated with a decrease in the number of dead neurons and the area of brain damage after ICH in mice. Moreover, MK3102 prominently upregulated glucagon-like peptide-1 receptor (GLP-1R) levels after ICH. In addition, the elevation of iron content, lipid peroxidation and FACL4 after ICH; and reduction of GPX4 and AIFM2; were mitigated by MK3102 in vitro and in vivo. The neuroprotective effect of MK3102 may be related to anti-ferroptosis by regulating GLP-1R after ICH injury.

## Introduction

Intracerebral hemorrhage (ICH) is a devastating disease with a 50% mortality rate within the first month, and with more than 75% of survivors being functionally disabled at 1 year^1^. The large hematoma is formed by blood penetrating into the brain parenchyma which mechanically compresses and tears brain tissue surrounding the lesion^2–4^. After the primary brain injury, various toxic substances are released by the hematoma and inflammatory cells resulting in severe inflammatory responses and oxidative stress; these lead to irreversible neuronal loss and neurological dysfunctions^5–7^.

Ferroptosis is an iron-dependent non-apoptotic cell death type identified by Dixon et al. in 2012^8^. It is characterized by over-load of iron, peroxidation of lipids and inactivation of antioxidant enzymes^5^. Large amounts of iron are released by degradation of hemoglobin/hemin after ICH and the consequent overload of iron in brain parenchyma is a key contributor of perihematomal cell death^9^. Recent evidence show that metamorphic mitochondria accumulate in cultured neurons after hemoglobin or hemin stimulation, and at the margin of the hematoma at 3 days after ICH in vivo^10^. These data indicate that ferroptosis is induced by ICH injury according to the morphological characteristics of ferroptosis^11^. In support, an inhibitor of ferroptosis, ferrostatin-1, markedly improves ICH-induced neurological deficits and memory impairment^5^.

Dipeptidyl peptidase-4 (DPP4), also known as CD26, is a membrane-associated peptidase encoded by mitochondria and widely distributed in body organs where it exerts pleiotropic effects via degrading target peptides such as glucagon-like peptide-1 (GLP-1), GIP and neuropeptide Y^12^. CD26 is a key enzyme associated with ferroptosis induction^13^. Blocking the activity of CD26 with inhibitors (vildagliptin, alogliptin, and linagliptin) antagonized ferroptosis induced by erastin in TP53-/- and TP53-depleted human colorectal cancer cells^14^.

Omarigliptin (MK3102) is a novel long-acting CD26 inhibitor with a long terminal half-life (ranging from 11 to 22 h) in preclinical species and it has great ability to block CD26 activity compared to other CD26 inhibitors^15^. Moreover, MK3102 can cross the blood–brain barrier (BBB) easily after oral administration due to its low molecular weight and lipophilic properties^16^. The high concentration of MK3102 in the brain significantly increases the level of GLP-1^17^, which further acts on GLP-1 receptors (GLP-1R) and exerts pleiotropic neuroprotective effects^18^. Previous studies report that MK3102 mitigates neuroinflammation induced by lipopolysaccharide^15^ and oxidative stress injury in Parkinson’s disease in rats^19^. However, whether ferroptosis would be alleviated by MK3102 after ICH injury remains unclear. In this study, we aimed to explore the anti-ferroptosis effect of MK3102 after ICH injury through a series of experiments in vitro and in vivo.

## Results

### MK3102 reduces the neurotoxicity of hemin in a concentration-dependent manner in vitro

MK3102 is a novel potent DPP4 inhibitor for treatment of type-2 diabetes mellitus^20^. It is becoming increasingly evident that MK3102 provides neuroprotection against neurodegenerative disease^19^. In our current study, the neuroprotective effect of MK3102 was explored in the pathological state of ICH injury.

**Figure 1:**
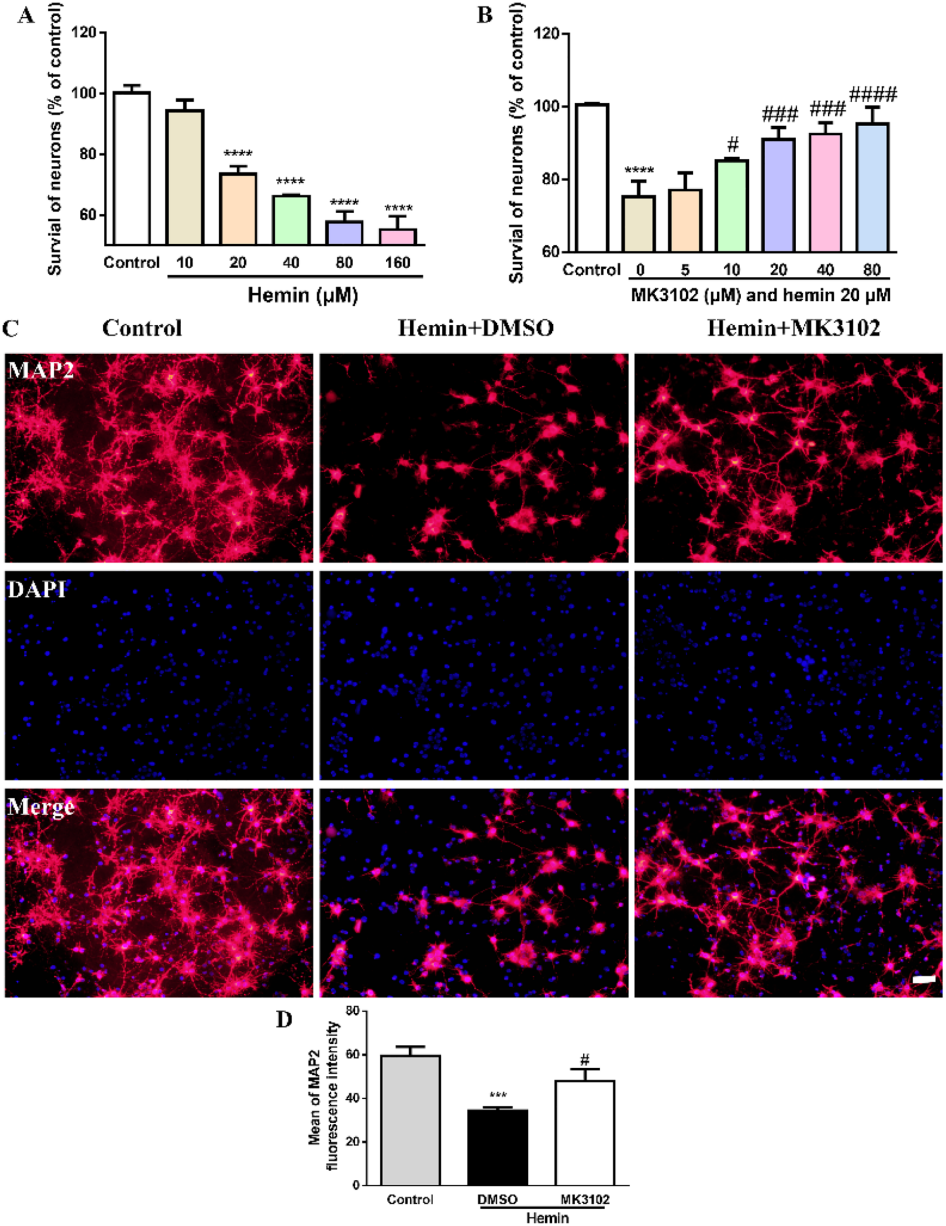
MK3102 reduces the neurotoxicity of hemin in cultured primary cortical neurons in a concentration-dependent manner. (**A**) Primary cortical neurons were subjected to various concentrations of hemin and cell viability was determined by CCK-8 assay. Hemin killed primary cortical neurons. (**B**) The toxicity of 20 μM of hemin was attenuated by MK3102 in a concentration-dependent manner. Results are expressed as the percent of neurons remaining in culture compared with that in control cultures. Each bar is the mean ± SD of triplicate wells. (**C**) Representative microphotographs of MAP2 positive cells in cultured primary cortical neurons. Scale bar, 50 μm. (**D**) Quantitative analysis of mean fluorescence intensity of MAP2 in cultured primary cortical neurons (n = 3 per group). All data are displayed as mean ± SD. The difference between groups was analyzed using One-way ANOVA test. ***p < 0.001, ****p < 0.0001, compared with control group. #p < 0.05, ^###^p < 0.0001, ^####^p < 0.0001, compared with hemin + DMSO group.

Cultured primary cortical neurons were treated by hemin with or without MK3102 for 24 h. Cell viability was then evaluated with CCK-8 assay. The toxicity of hemin was found to be concentration-dependent, and more than 20 μM of hemin resulted in noticeable loss of primary cortical neurons (Fig. 1A).

Using 20 μM of hemin to simulate neuronal injury in vitro, we next measured the protective capacity of MK3102. The cell viability of neurons was elevated by MK3102 in a concentration-dependent manner (Fig. 1B). MAP2 staining demonstrated that the degeneration of neurons by hemin was attenuated by MK3102 (Mean of MAP2 fluorescence intensity in Hemin + MK3102 group, 47.98 ± 5.511 versus Hemin + DMSO group, 34.46 ± 1.343, p < 0.05, Fig. 1C). These results confirm that MK3102 has a neuroprotective effect on primary cortical neurons subjected to hemin toxicity. According to the above results, 20 μM hemin and 10 μM MK3102 were chosen as the optimal concentrations for subsequent studies.

### MK3102 provides neuroprotection after ICH in mice

**Figure 2:**
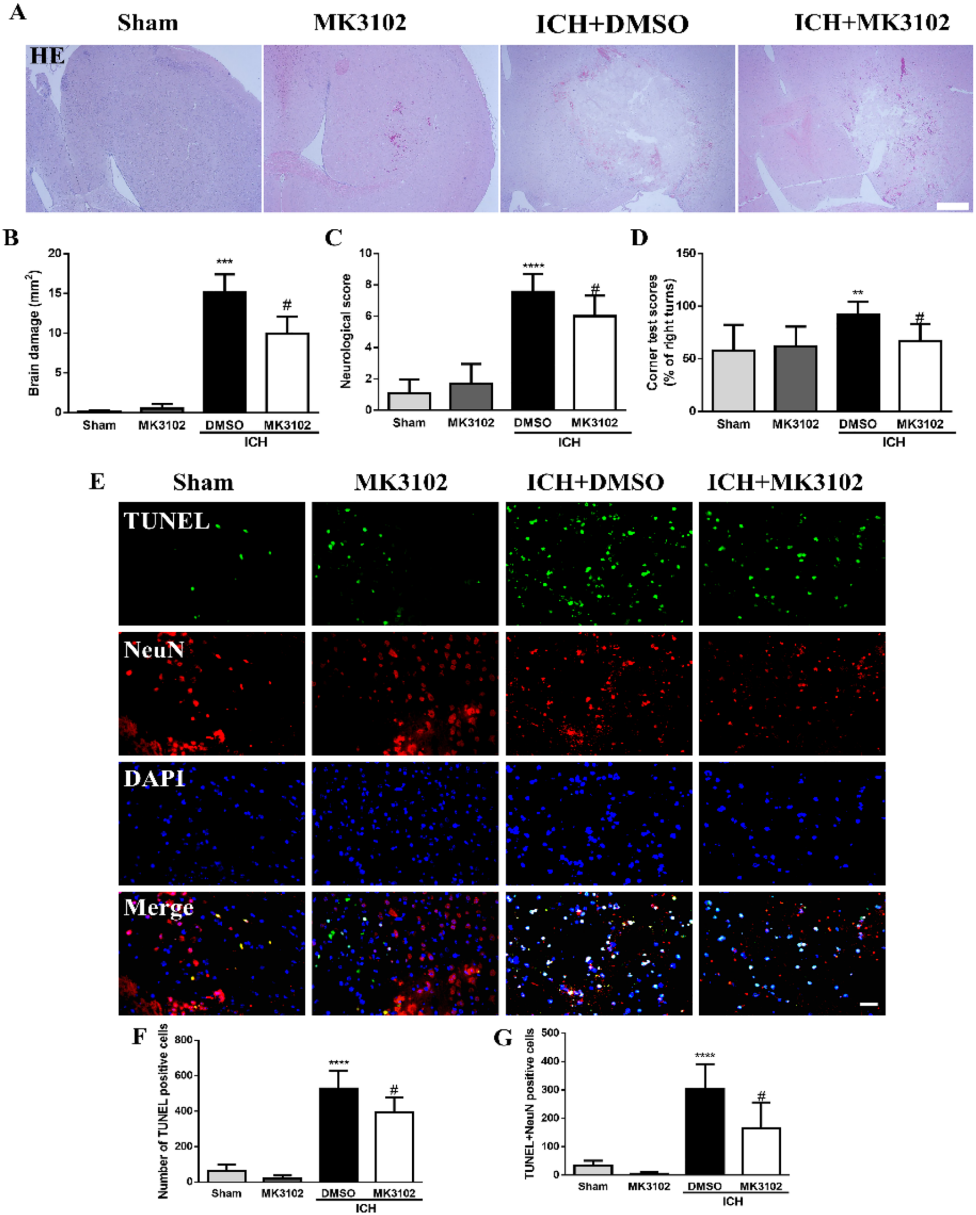
MK3102 treatment alleviates brain damage after ICH in mice. (**A**) Representative microphotographs of HE staining at 3 days after ICH. Scale bar, 200 μm. (B) Quantitative analysis of brain damage area (n = 3 per group). (**C**,**D**) Quantitative analysis of neurological scores and corner test scores at 3 days after ICH (n = 10 per group). (**E**) Representative microphotographs of TUNEL positive cells, NeuN positive cells, TUNEL and NeuN double positive cells in the right brain hemisphere. Note that NeuN staining was not uniform in the sham and MK3102 group tissue because of some degree of injury caused by the sterile saline injection. Scale bar, 20 μm. (**F**,**G**) Quantitative analysis of TUNEL positive cells and TUNEL positive neurons in the right brain hemisphere at 3 days after ICH (n = 5 per group). All data are displayed as mean ± SD. The difference between groups was analyzed using One-way ANOVA test. **p < 0.01, ***p < 0.001, ****p < 0.0001 compared with sham and MK3102 group. ^#^p < 0.05 compared with the ICH + DMSO group.

To explore whether MK3102 had neuroprotective effects in mice with ICH, the behavioral, pathological and molecular biological changes of 96 mice were analyzed in this study. HE staining showed that MK3102 treatment significantly decreased brain damage area 3 days after ICH when compared with ICH + DMSO treated group (9.937 ± 2.137 mm^2^ versus 15.15 ± 2.258 mm^2^, p < 0.05, Fig. 2A,B).

Behavioral assessments showed that MK3102 had no effect on the neurobehavioral performance of non-injured mice. Much higher values of neurological scores and the corner tests were observed after ICH injury. However, both of them were significantly reduced by MK3102 at a dose of 7 mg/kg per day at 3 days following ICH injury when compared with ICH + DMSO treatment group (6.000 ± 1.309 versus 7.556 ± 1.130 of neurological scores, p < 0.05; 67.00 ± 16.36% versus 92.00 ± 12.29% of corner tests, p < 0.05, Fig. 2C,D).

Encouraged by these results, we further examined whether brain cell death would be lowered by MK3102 intervention. We used TUNEL staining to identify dead cells with damaged DNA. In the sham and MK3102 group, only a few TUNEL-positive cells were observed in the right brain hemisphere. However, compared with the sham and MK3102 group, a large number of TUNEL and TUNEL/NeuN positive cells appeared in the right brain hemisphere of the ICH group. MK3102 treatment markedly reduced the number of TUNEL and TUNEL/NeuN positive cells compared with that in the ICH + DMSO group (391.4 ± 87.48 versus 528.0 ± 101.9, p < 0.05; 164.2 ± 92.41 versus 303.0 ± 88.33, p < 0.05, respectively, Fig. 2E–G). These results suggest that MK3102 has a neuroprotective effect on ICH mice.

### MK3102 regulates GLP-1R after ICH in vivo

**Figure 3:**
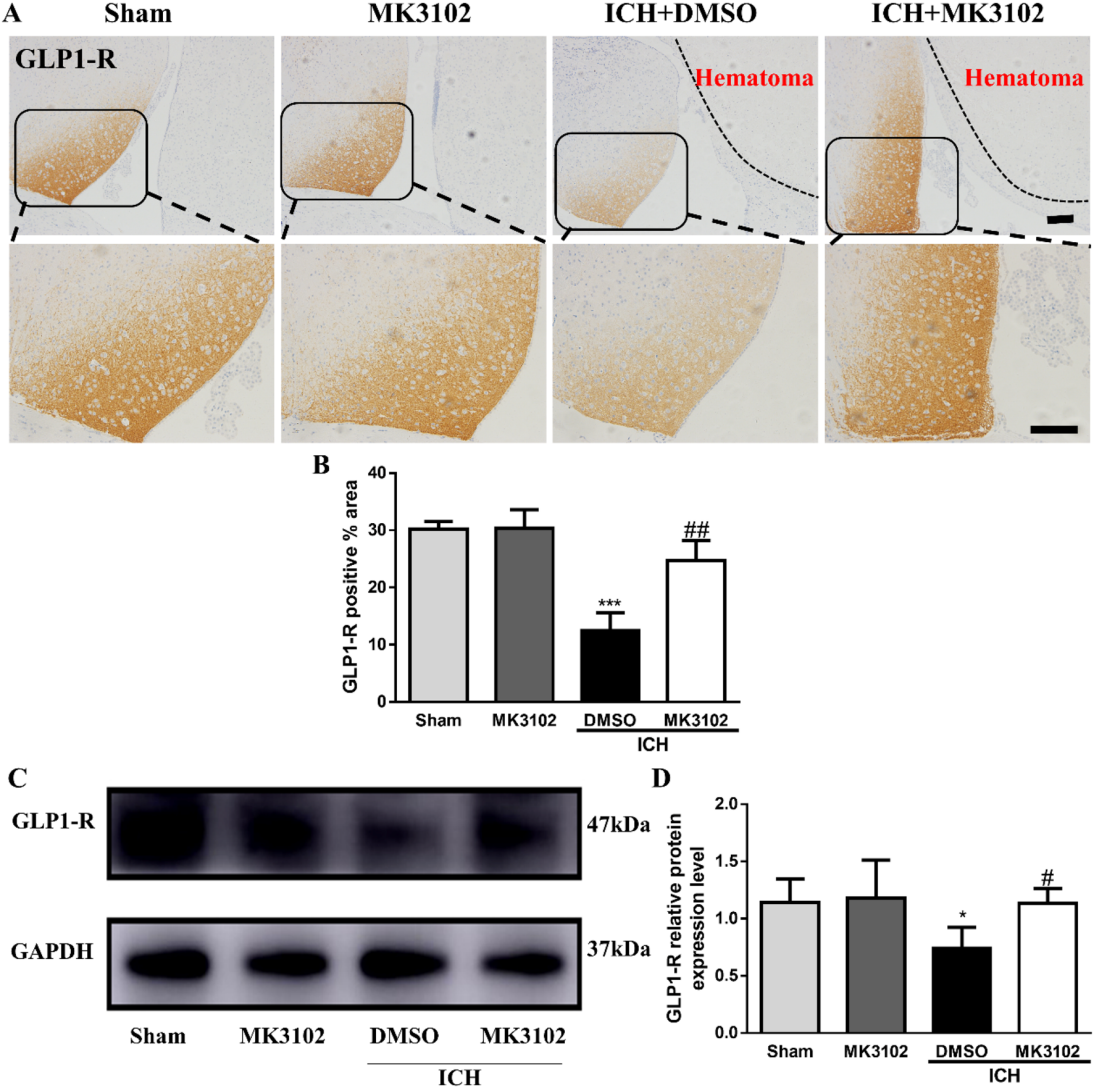
MK3102 attenuates the toxicity of collagenase on GLP-1R in mice. (**A**) Representative immunohistochemistry images of GLP-1R in the right brain hemisphere at 3 days after ICH. Short scale bar, 100 μm; Long scale bar, 50 μm. (**B**) Quantitative analysis of GLP-1R positive area. n = 3 per group. (**C**) Representative Western blot bands of GLP-1R. Original blots are presented in Supplementary Fig. 1. (**D**) Quantitative analyses relative protein expression level of GLP-1R in the right brain hemisphere at 3 days after ICH. n = 6 per group. All data are displayed as mean ± SD. The difference between groups was analyzed using One-way ANOVA test. *p < 0.05, ***p < 0.001 compared with sham and MK3102 group. ^#^p < 0.05, ^##^p < 0.01 compared with the ICH + DMSO group.

Accumulating evidence demonstrate that inhibition of CD26 with MK3102 significantly increases the activity of GLP-1, which then produces potent anti-oxidative stress effects through binding to GLP-1R^19^. We hypothesize that the neuroprotective effect of MK3102 may be associated with regulating the CD26/GLP-1/GLP-1R axis following ICH. Our immunohistochemical results showed that a high % of GLP-1R immunoreactive area was observed in the right brain hemisphere of sham mice; notably, this was significantly reduced following ICH. Compared with the vehicle group, MK3102 treatment dramatically increased the % of GLP-1R immunoreactive (24.71 ± 3.538 versus 12.48 ± 3.102, P < 0.01, Fig. 3A,B). We obtained corroborative results by western blotting analysis (1.133 ± 0.1314 versus 0.7420 ± 0.1820, P < 0.05, Fig. 3C,D). These results suggest that the DPP4 inhibitor MK3102, which potently blocks DPP4, may protect neurons from ICH injury by regulating the activity of GLP-1R.

### MK3102 reduces ferroptosis in cultured primary cortical neurons in vitro

**Figure 4:**
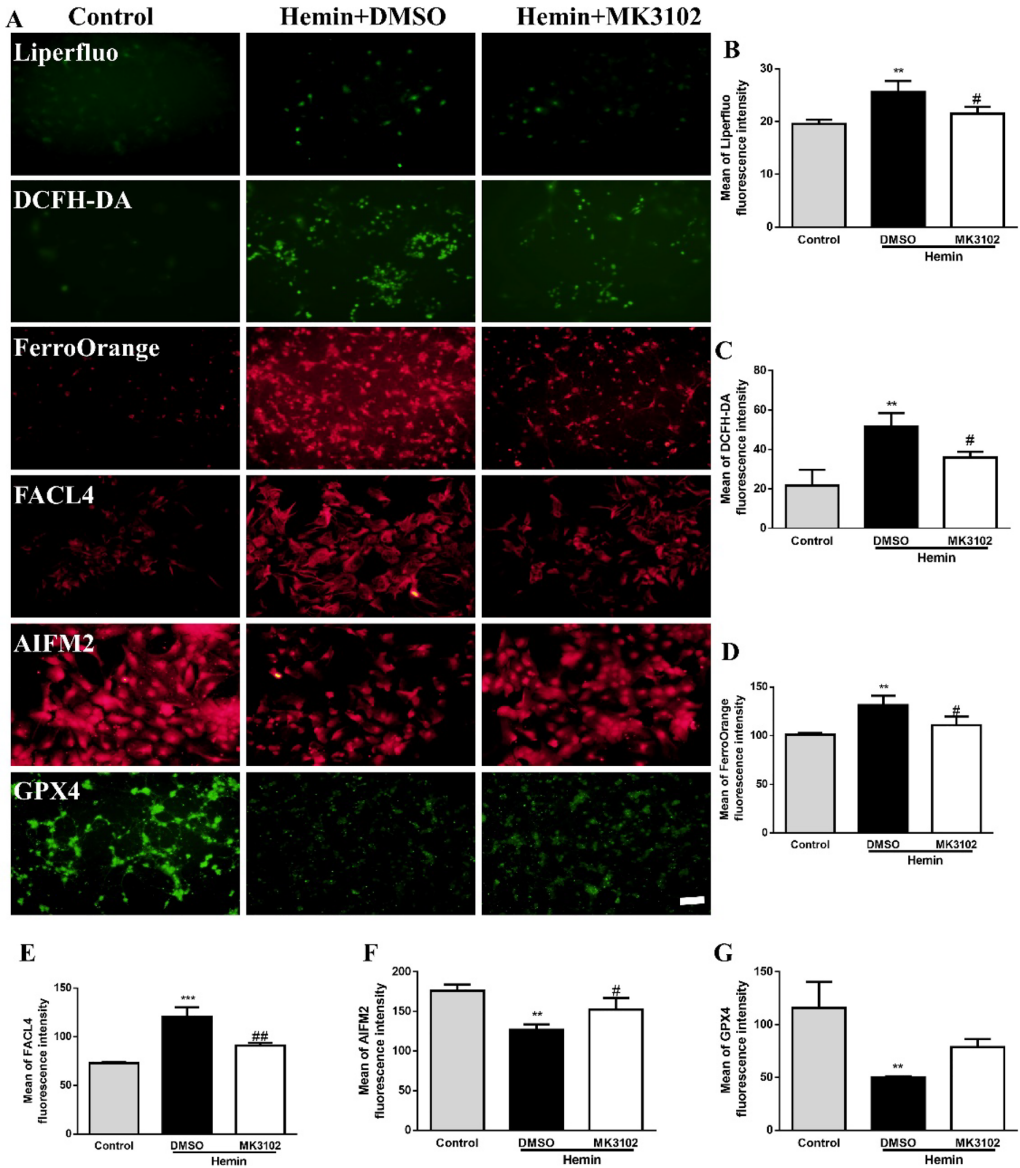
MK3102 reduces hemin induced ferroptosis in cultured primary cortical neurons. (**A**) Representative fluorescent microphotographs of Liperfluo, DCFH-DA, FerroOrange, FACL4, AIFM2, and GPX4 in cultured primary cortical neurons. Scale bar, 50 μm. (**B**–**G**) Quantitative analysis of mean fluorescence intensity of Liperfluo, DCFH-DA, FerroOrange, FACL4, AIFM2, and GPX4 in cultured primary cortical neurons (n = 3 per group). All data are displayed as mean ± SD. The difference between groups was analyzed using One-way ANOVA test. **p < 0.01, ***p < 0.001, ****p < 0.0001 compared with control group. ^#^p < 0.05, ^##^p < 0.01, ^####^p < 0.0001, compared with hemin + DMSO group.

In this study, intracellular reactive oxygen species (ROS), iron and lipid peroxidation levels were determined by DCFH-DA, FerroOrange, and Liperfluo in cultured primary cortical neurons 8 h after hemin treatment. We found much higher levels of ROS, iron and lipid peroxidation in the hemin compared to control group. Concurrently, compared with control, the content of Acyl-CoA synthetase long-chain family member 4 (FACL4), a lipid metabolism enzyme which promotes ferroptosis, was significantly increased while the antioxidant protein glutathione peroxidase 4 (GPX4) and apoptosis inducing factor mitochondria associated 2 (AIFM2) were markedly decreased in the hemin group. However, treatment with MK3102 compared to vehicle significantly reduced the levels of ferroptosis markers (DCFH-DA, FerroOrange, Liperfluo and FACL4), and increased the expression of anti-ferroptosis markers (GPX4 and AIFM2) after hemin administration (Fig. 4). These results further corroborate that MK3102 protect neurons from ICH injury probably via defending against ferroptosis in vitro.

### MK3102 inhibits brain cell ferroptosis after ICH injury in mice

**Figure 5:**
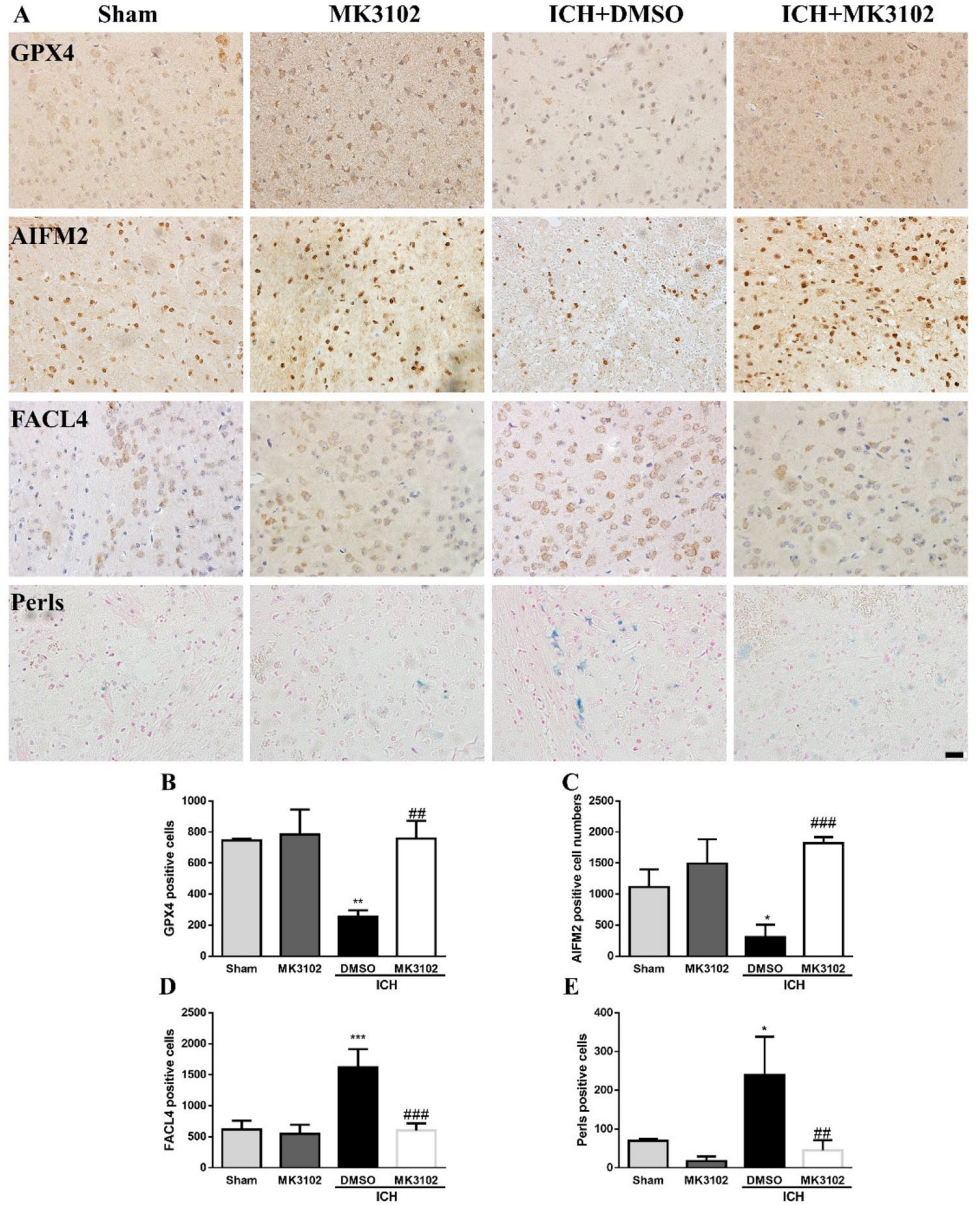
MK3102 inhibits brain cell ferroptosis after ICH in mice. (**A**) Representative immunohistochemistry images of GPX4, AIFM2, FACL4, and Perls staining in the right brain hemisphere at 3 days after ICH. Scale bar, 20 μm. (**B**–**E**) Quantitative analysis of GPX4, AIFM2, FACL4, and Perls positive cells. n = 3 per group. All data are displayed as means ± SD. The difference between groups was analyzed using One-way ANOVA test. *p < 0.05, **p < 0.01, ***p < 0.001, compared with sham and MK3102 group. ^##^p < 0.01, ^###^p < 0.001 compared with the ICH + DMSO group.

Our immunostaining results showed that the expression levels of GPX4 and AIFM2 were downregulated, while FACL4 and iron deposition were markedly increased, after ICH injury, which indicated that ferroptosis was induced following ICH in mice. Interestingly, compared to the ICH + DMSO group, MK3102 significantly decreased the expression levels of FACL4 and iron deposition while it elevated the expression of GPX4 and AIFM2 after ICH injury (45.33 ± 25.72 versus 239.0 ± 99.18 for FACL4, p < 0.01; 602.3 ± 116.1 versus 1619.0 ± 294.6 for iron, p < 0.001; 758.0 ± 113.8 versus 256.7 ± 38.73 for GPX4, p < 0.01; 1821 ± 96.47 versus 304.7 ± 205.7 for AIFM2, p < 0.001, respectively, Fig. 5). These findings infer that MK3102 inhibits brain cell ferroptosis after ICH injury in mice.

## Discussion

MK3102 is a potent DPP4 inhibitor for type-2 diabetes patients^21^. Interestingly, in addition to its anti-hyperglycemic effects, MK3102 exerts anti-inflammatory activities and protects the integrity of the BBB from lipopolysaccharide probably via inhibiting the activation of the toll-like receptor 4 (TLR4)/myeloid differentiation factor 88/nuclear factor kappa B (NF-κB) signaling pathway^15^. Moreover, it also attenuates oxidative stress injury in a variety of pathological states^19,20^. For example, cognitive dysfunction was alleviated with MK3102 treatment in Streptozotocin-induced diabetic mouse^22^. In this study, the survival rate of cultured neurons was improved by MK3102 in a concentration-dependent manner after hemin treatment (Fig. 1), and neurological dysfunction after ICH in mice was alleviated associated with reduction of area of brain injury and neuron loss (Fig. 2). These results suggest that MK3102 confers neuroprotective effect probably through various molecular signaling mechanisms.

GLP-1 is the main substrate of CD26 which can be completely and reversely inhibited by MK3102^23^. GLP-1 comes from the gut and brain and acts on GLP-1 receptors which are widely expressed in the hippocampus, cortex, choroidal plexus and other brain regions to exert central anti-oxidative stress ability in ischemic stroke, Parkinson’s disease and Alzheimer disease^24^. Our results show that GLP-1R was significantly decreased following ICH in mice and that MK3102 prevented this downregulation (Fig. 3). These data indicate that the neuroprotective effects of MK3102 are associated with its regulation of GLP-1R after ICH.

Following ICH, large amounts of iron are released by the degradation of hemoglobin/hemin in the hematoma. Iron may react with H_2_O_2_ to produce **·**OH (a kind of ROS) through the Fenton reaction in brain cells^25^. The highly toxic **·**OH can result in oxidative damage in cell structures or components such as carbohydrates, nucleic acids, lipids and proteins^9^. Lipid peroxidation plays a key role in driving ferroptosis^26^. This process is promoted by FACL4^27^, and blocked by GPX4 through reducing phospholipid hydroperoxides and AIFM2 by catalyzing reduction of coenzyme Q/ubiquinone-10 to ubiquinol-10 to eliminate lipophilic radicals that prevent lipid oxidative damage and consequently ferroptosis^28^.

Our previous research noted that the level of malondialdehyde (a lipid peroxide) and iron deposition was significantly increased but GPX4 expression was markedly reduced after ICH in mice^12^. Jin et al. demonstrated that the expression level of FACL4 in HT22 cells or cortical neurons was markedly elevated while GPX4 was inhibited by hemin administration in vitro^29^. Consistent with these results, our research showed that much higher level of Fe^2+^ (determined by FerroOrange) or Fe^3+^ (observed by Perls staining), ROS (detected by DCFH-DA), lipid peroxidation (shown by Liperfluo) and FACL4 (indicated by immunohistochemistry or immunofluorescence) were detected after ICH injury compared to control or sham group, while lower expression of GPX4 and AIFM2 occurred (Figs. 4 and 5). These results suggest that ferroptosis is triggered by ICH injury, which causes neuronal loss and neurobehavioral dysfunction. Increasing evidence demonstrate that ferroptosis is a major contributor of neuronal death after ICH, and inhibition of neuronal ferroptosis may effectively reduce secondary brain damage after ICH^30^.

It has been suggested that GLP-1R exerts its effect through a pre-synaptic enhancement of glutamate release in the brain^31^. However, the released glutamate may activate the cystine-glutamate antiporter system X_C_^-^ to synthesize GPX4 by exchanging cystine in a 1:1 ratio^8^. Importantly, CD26 promotes lipid peroxidation through NADPH oxidase^32^. Thus, we speculated that MK3102 may inhibit ferroptosis by degrading CD26 and elevating GLP-1/GLP-1R levels after ICH injury. Surprisingly, MK3102 treatment reversed the increased iron deposition, lipid peroxidation, FACL4, and the reduction of GPX4 and AIFM2 after ICH injury in vitro and *vivo* (Figs. 4 and 5). Therefore, our results reveal that the neuroprotective effects of MK3102 may be related to anti-ferroptosis by regulating the CD26/GLP-1/GLP-1R axis after ICH injury.

Several limitations in our study are also worth noting. Here, we only tested the neuroprotective effect of MK3102 in the acute phase of ICH pathology; more investigations are needed to confirm whether it also has a benefited effect on the subacute and chronic phases of ICH injury. Moreover, the downstream molecular mechanism of ferroptosis inhibition by MK3102 is unclear, and more research is needed to explore the molecular pathways. In addition, we only tested it on male mice because previous studies have shown that estrogen has neuroprotective effect in ICH in animals^33^; thus, the protective effect of MK3102 in females after ICH deserves investigation. Another limitation is that the direct morphological characteristics of ferroptosis, such as mitochondrial fragmentation and cristae enlargement, were not evaluated by transmission electron microscope in this study. Lastly, we cannot rule out that other molecular pathways also mediate the neuroprotective effects of MK3102, such as apoptosis and inflammation.

## Conclusion

In summary, our findings demonstrate that MK3102 has neuroprotective effects in vitro and *vivo* following ICH. MK3102 may inhibit brain cell ferroptosis by regulating GLP-1R after ICH injury. Our study provides a new perspective for the clinical translational application of MK3102 in ICH.

## Materials and methods

### Experimental animals

A total of 102 C57BL/6 mice (96 mice aged 8 weeks for in vivo animal experiment and 6 litters of neonatal mice aged 1–2 days for in vitro experiment) were purchased from Beijing Vital River Experimental Animals Centre (Beijing, China) and maintained in a Specified Pathogen Free room with temperature at 22 °C, relative humidity at 55%, and light/dark cycle at 12/12 h. All experimental procedures were approved by the Ethics Committee of Zhengzhou University, according to the Laboratory Animal—Guideline for Ethical Review of Animal Welfare. The present study is reported in accordance with ARRIVE guidelines.

### Cultivation of primary cortical neurons and treatment administration

Primary cortical neurons were extracted from the cerebral cortex of 1–2 day(s) neonatal mice, as described previously^34^. In brief, mice were anesthetized by CO_2_ inhalation. Cortical tissues were isolated on ice under aseptic conditions after removing the meninges and blood vessels, and then digested with 0.25% trypsin and 0.001% DNase I (Invitrogen, Carlsbad, CA, USA) at 37 °C for 15 min. Cell suspensions were respectively seeded in 24-well plates and 96-well plates coated with 0.01% poly-L-lysine (Solarbio, Beijing, China) at a density of 2 × 10^5^ cells/well and 2 × 10^4^ cells/well, and cultured in Neurobasal medium containing 1% Glutamax and 2% B27 (Invitrogen, Carlsbad, CA, USA) for 9 days. Cells were then treated with their respective drugs while control cells were exposed only to culture medium. Neurons in the hemin + DMSO group were exposed to medium with added hemin and 0.1% DMSO as vehicle; while cells in the hemin + MK3102 group were exposed to medium with added hemin and MK3102 (in 0.1% DMSO). MK3102 was added to the medium immediately after hemin administration. The cells were used for fluorescent staining and cell viability analysis at preselected times after hemin stimulation.

### Cell viability assay

Cell viability was evaluated with Cell Counting Kit-8 (CCK-8, Glpbio, USA). After treatment, CCK-8 solution was added to medium in a ratio of 1:9. The cells were then cultured in the incubator for 3 h, and the absorbance at 450 nm was measured by SpectraMax M5/M5e (Molecular Devices, USA). Cell viability was expressed as a percentage of that in the control group.

### The ICH model and MK3102 administration

Mice were anesthetized with an isoflurane-oxygen mixture during the surgical procedure and the head was immobilized using a brain stereotactic apparatus (RWD, Shenzhen, China). The coordinates of the target (the midline 2.0 mm to the right and the bregma 0.2 mm to the posterior) were determined according to the stereoscopic map of the mouse brain. After the skull was punctured by electric drill, the needle (Hamilton, Switzerland) was inserted to a depth of 3.5 mm under the skull, and 0.075 U of collagenase type VII (Sigma-Aldrich, USA) was injected into the target at a rate of 0.1 μL/minute. After injection, the needle was maintained for 10 min to prevent reflux, and the micro syringe needle was slowly withdrawn. Bone wax was used to seal the burr hole. The wound was then sutured and disinfected. All mice had free access to food and water after surgery.

All mice were randomly assigned to four groups: sham group, MK3102 group, ICH + DMSO treated group, and ICH + MK3102 treated group. The coordinates of the target in ICH mice received 0.075U collagenase, while sham and MK3102 group were given the same volume of sterile saline. For the MK3102 treatment, the MK3102 (Glpbio, USA) was dissolved in 10% DMSO and administrated (7 mg/kg/day) by gastric injection starting from 1 h after surgery. The dosage of MK3102 was determined by the conversion of rat doses to mouse equivalent doses based on body surface area and the 11 ~ 22 h half-life pharmacokinetic characteristics of MK3102^15,16^. The ICH + DMSO group was received the same volume of DMSO. All treatments were conducted for 3 days as our previous work demonstrated that prominent pathology of ICH was observed by 3 days after collagenase type VII injection^12^.

### Behavioral assessments

Behavioral assessments were carried out by two independent investigators blinded to the experimental groups using the focal deficits neurological scores and corner test before the mice were sacrificed.

Focal deficits neurological scores: the Focal deficits neurological scores system consists of 7 items via evaluating limb movement and sensation. Each item is divided into 0 to 4 points according to different performance, with a total score of 28 points. The higher the score, the more severe brain injury^35^.

Corner turn test: limb function of mice was detected by corner turn test. Mice were placed in a corner of 30-degree, and the percentage of right turns over 10 repeats was recorded.

### HE staining

Hematoxylin and eosin (HE) staining was used to evaluate areas of brain damage, which were defined by the presence of blood, tissue rarefaction or necrosis. The sections were placed in the hematoxylin solution for 5 min and then incubated with eosin for 2 min. Pictures of HE stained sections were taken with Olympus microscope (Olympus Co., Japan) by using 4X objective. The brain damage areas were analyzed with Image Pro-Plus 6.0 software, and the sum from the three sections of the right brain hemisphere represents the area of brain damage per mouse.

### NeuN immunofluorescent and terminal deoxynucleotidyl transferase-dUTP nick-end labeling (TUNEL) double staining

TUNEL (Vazyme Biotech, Nanjing, China) and NeuN were performed to quantify neuronal death after ICH. Briefly, the sections embedded in paraffin were dewaxed and rehydrated with gradient alcohol, and then blocked with blocking buffer with Triton X-100 (Beyotime, Nanjing, China) for 1 h and incubated with FITC-12-dUTP labeled mixture at 37 °C for another hour. After washing with PBS, the sections were incubated overnight under 4 °C with antibody against NeuN (1:100, Abcam, Cambridge, MA, USA). The TUNEL and NeuN positive cells were observed with Olympus microscope (Olympus Co., Japan) by using 40X objective. The number of positive cells in four fields per section from three sections of the right brain hemisphere was counted manually with Image Pro-Plus 6.0 software and summed for each mouse.

### Ferroptosis evaluation

#### Intracellular ROS, iron, and lipid peroxidation measurement

DCFH-DA, FerroOrange, and Liperfluo were performed to determine the intracellular ROS, iron, and lipid peroxidation levels, respectively^36^. The treated primary cortical neurons were stained with 10 μM of DCFH-DA, 1 μM FerroOrange, and 20 μM Liperfluo for 30 min in the dark at 37 °C, respectively. The fluorescence in 24-well plates were visualized under an inverted fluorescent microscope (Olympus Co., Japan). Image Pro-Plus 6.0 was used to analyze the mean of fluorescent intensity in 4 fields per well.

#### Perls staining

Perls staining was used to visualize Fe^3+^ deposition after ICH. Prepared sections were immersed in Perls solution (Solarbio, Beijing, China) for 30 min. After rinsing with distilled water, the sections were counterstained with Nuclear Fast Red Solution for 5 min. The positive cells were observed with Olympus microscope (Olympus Co., Japan) using 40X objective. The number of positive cells in four fields per section from three sections of the right brain hemisphere was counted manually with Image Pro-Plus 6.0 software and summed for each mouse.

#### Immunohistochemistry

After various treatments, brains were harvested, paraffin-embedded and then cut into 5 μm thick sections. Sections from the hematoma center and 200 μm on both sides of the hematoma center were used for analysis. Briefly, sections were incubated under 4 °C overnight with primary antibodies against rabbit anti-AIFM2 polyclonal antibody (1:100, Affinity Biosciences, OH, USA), mouse anti-GPX4 monoclonal antibody (1:50, Santa Cruz Biotechnology, CA, USA), rabbit anti-GLP-1R monoclonal antibody (1:100, Abcam, Cambridge, MA, USA), or rabbit anti-FACL4 monoclonal antibody (1:100, Abcam, Cambridge, MA, USA). The sections were then incubated with horseradish peroxidase-combined secondary anti-rabbit or mouse immunoglobulin G antibodies (1:1000, Abcam, Cambridge, MA, USA) for 1 h at room temperature. All staining outcomes were observed using Olympus microscope (Olympus Co., Japan), and the positive cells or area in 4 fields per tissue section from the right brain hemisphere were analyzed with Image Pro-Plus 6.0 software. Analyses were conducted in a blinded manner.

#### Immunofluorescence

The paraffin embedded brains were prepared on the third day after ICH with the method described above. For the primary cortical neurons, the cells were seeded into 24-well culture plate at 2 × 10^5^ cells/well for immunofluorescent staining. Tissue sections and cells were incubated overnight at 4 °C with rabbit anti-MAP2 monoclonal antibody (1:2000, Abcam, Cambridge, MA, USA), rabbit anti-NeuN monoclonal antibody (1:100, Abcam, Cambridge, MA, USA), rabbit anti-AIFM2 polyclonal antibody (1:100, Affinity Biosciences, OH, USA), mouse anti-GPX4 monoclonal antibody (1:50, Santa Cruz Biotechnology, CA, USA), or rabbit anti-FACL4 monoclonal antibody (1:100, Abcam, Cambridge, MA, USA). After PBS rinsing, the samples were incubated in the corresponding Alexa Fluor-conjugated secondary antibody (1:500, Abbkine, USA) for 1 h in the dark at room temperature. The anti-fluorescence quenching mounting medium containing DAPI (Solarbio, Beijing, China) was added to the sample area. Then all sections and cells were observed under fluorescence microscope and the positive cells or mean fluorescent intensity in 4 fields per section/wells were counted with Image Pro-Plus 6.0 software.

### Western blot

Mice were euthanized at 3 days after ICH and brain homogenate was extracted from the entire right brain hemisphere of each mouse. Western blot was performed as described previously^12^. Briefly, total protein was extracted using RIPA buffer with 1% PMSF (Solarbio, Beijing, China). After quantitative analysis of the total protein concentration with BCA method, the samples were separated by 10% SDS-PAGE and transferred to PVDF membrane (Millipore, USA). To separate the region where the target protein will appear, the membrane was cut along the molecular weight marker. Then the PVDF membrane was blocked with Blocking Buffer (Beyotime, Nanjing, China) at room temperature for 1 h and incubated at 4 °C on a shaker with the anti-GLP-1R (1:1000, Abcam, Cambridge, MA, USA)^37^ and anti-GAPDH (1:2000, Servicebio, China) antibodies. After washing with TBST, the membrane was placed in the configured secondary antibodies (Goat anti-mouse/Rabbit HRP, 1:5000), incubated at room temperature for 1 h. The immunoblot was visualized with Ultra High Sensitivity ECL Kit (GlpBio, Montclair, USA) through Amersham Imager 600. The relative intensity of the bands was measured by ImageJ (NIH, USA).

#### Statistical analysis

Statistical analyses were performed by the GraphPad Prism Version 6.0 (GraphPad, La Jolla, CA, USA). All data obtained were presented as mean ± standard deviation (SD) of at least three independent experiments. The Shapiro–Wilk test and/or D'Agostino & Pearson omnibus normality test were used for the data normality test. Comparisons between multiple groups were made using One-way analysis of variance (ANOVA), followed by Tukey’s Honestly Significant Difference (HSD) post-hoc test. The Kruskal–Wallis test was used to analysis the data that did not exhibit the normal/ Gaussian distribution. P < 0.05 was defined as statistically significant.

### Ethics statement

All experimental procedures were approved by the Ethics Committee of Zhengzhou University (2021326). National Standards of the People’s Republic of China (GB/T 35892–2018), Laboratory Animal—Guideline for Ethical Review of Animal Welfare, was the guidance for our animal care and protocols.

### Supplementary Information

Supplementary Figure 1.

## Data Availability

The original data generated in this study are included in the article/supplementary material, further inquiries can be directed to the corresponding authors.
