# Supplementary material for: Omarigliptin inhibits brain cell ferroptosis after intracerebral hemorrhage
Source: Sci Rep. 2023 Sep 1;13:14339. doi: 10.1038/s41598-023-41635-y (PMC10474264; doi:10.1038/s41598-023-41635-y)
Supplement: Supplementary file 1 — Supplementary Figure 1. [file 41598_2023_41635_MOESM1_ESM.pdf]

Supplementary Information

Omarigliptin inhibits brain cell ferroptosis after intracerebral hemorrhage

Author: Yan Zhang[1,2], Yang Liu[1,2], V. Wee Yong[3], Mengzhou Xue[1,2]

[1] Department of Cerebrovascular Diseases, The Second Affiliated Hospital of Zhengzhou University, Zhengzhou, Henan, China

[2] Academy of Medical Science, Zhengzhou University, Zhengzhou, Henan, China

[3] Hotchkiss Brain Institute and Department of Clinical Neurosciences, University of Calgary, Calgary, Alberta, Canada

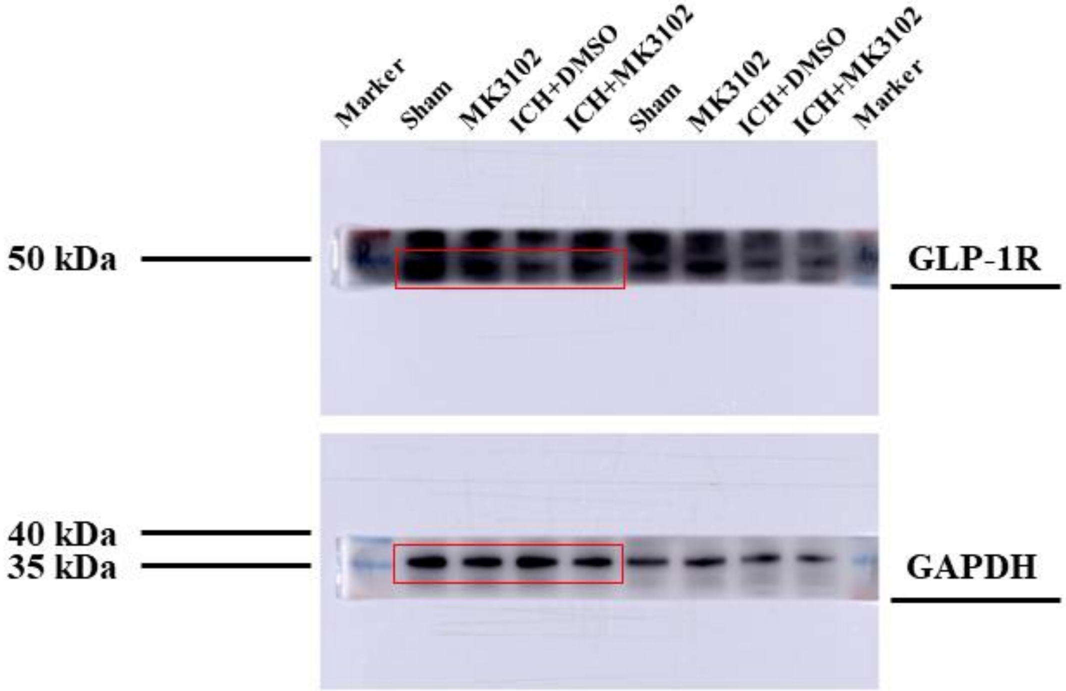

Supplementary Figure 1. Original Western blot blots of Figure 3 C (red box circled). Western blots were cropped prior to incubation with primary antibody hybridization.
